# Retraction: LncRNA NEXN-AS1 attenuates proliferation and migration of vascular smooth muscle cells through sponging miR-33a/b

**DOI:** 10.1039/d2ra90099h

**Published:** 2022-10-07

**Authors:** Leiming Wu, Yapeng Li, Dianhong Zhang, Zhen Huang, Binbin Du, Zheng Wang, Lulu Yang, Yanzhou Zhang

**Affiliations:** Department of Cardiology, The First Affiliated Hospital of Zhengzhou University No. 1 Jianshe East Road Zhengzhou 450052 Henan China chizi965014mb@126.com +86-37167967661

## Abstract

Retraction of ‘LncRNA NEXN-AS1 attenuates proliferation and migration of vascular smooth muscle cells through sponging miR-33a/b’ by Leiming Wu *et al.*, *RSC Adv.*, 2019, **9**, 27856–27864, https://doi.org/10.1039/C9RA06282C.

The Royal Society of Chemistry hereby wholly retracts this *RSC Advances* article due to concerns with the reliability of the data. The authors were asked to provide the raw data for the article, but stated they were unable to locate the raw data and asked to retract the article.

The western blots in Fig. 4F and 6D have been over-contrasted to the point where the background has almost been erased. In addition, there appear to be splice marks in the MMP-2 and β-actin panels in Fig. 7D, indicating that the images may have been manipulated. The authors are unable to provide verifiable raw data that could be used to validate the published images.

Given the significance of the concerns about the validity of the data, and the lack of raw data, the findings presented in this paper are not reliable.

The authors have not responded to any subsequent correspondence regarding the wording of the retraction notice.

Signed: Laura Fisher, Executive Editor, *RSC Advances*

Date: 18th August 2022

## Supplementary Material

